# Editorial: T Cell Differentiation and Function in Tissue Inflammation

**DOI:** 10.3389/fimmu.2020.00289

**Published:** 2020-02-21

**Authors:** Ritobrata Goswami, Amit Awasthi

**Affiliations:** ^1^School of Bioscience, Indian Institute of Technology Kharagpur, Kharagpur, India; ^2^Translational Health Science and Technology Institute, Faridabad, India

**Keywords:** CD4 T cell, CD8 T cell, cancer, autoimmune disease, inflammation

T cells constituting one of the arms of adaptive immune responses provide cell-mediated immunity against offending pathogens. Thymus is the maturation site for T cells that have been shown to be involved in cell-mediated immunity and humoral immune response in 1961–1962. It took another couple of decades to identify heterodimeric T cell receptor, which is crucial for the T cell activation, differentiation, and functions (1). In the next 25–30 years, several groundbreaking studies have contributed to the overall impact of T cells in modulating immune responses in health and diseases. T cell differentiation is one the key events that is absolutely essential for not only eliminating intra and extracellular pathogens but, upon dysregulation, could also lead to the onset of inflammation with exacerbate disease pathogenesis in autoimmune diseases. This Research Topic was developed to understand the complexity and molecular pathways that lead to the differentiation of Th cells that causes pathogenesis of disease. Under this Research Topic, a series of articles were published, which provided meaningful insights toward this emerging field. Briefly, this special issue is comprised of 8 original research papers, 5 full-length reviews, 3 mini-reviews, and 1 perspective to discuss the impact of T cell activation and differentiation in tissue inflammation. The original research articles included the role of CD4+ T cells in the pathophysiology of non-infectious uveitis and Graves' disease. The multi-faceted role of various subsets of CD4+ T cells have been reviewed extensively in tissue homeostasis, inflammatory bowel disease, osteoporosis, and neuroinflammation. These articles strongly support and provide new insight that harness the knowledge of Th cell differentiation may uncover novel therapeutic strategies to control inflammatory diseases.

While CD4+ T cells work by releasing cytokines, CD8+ T cells are cytotoxic. A recent study has fueled the notion that CD8+ T cells might be important factor for longevity (2). Adoptive T cell treatment has shown immense potential to train the immune system in fighting against deadly diseases such as cancer. Tumor-specific CD8+ T cells are inserted into patients that target and attack cancer cells. There are clinical trials that have shown successful outcome in treating metastatic melanoma using adoptive T cell therapy. Patient T cells have been genetically modified with synthetic receptors generating chimeric antigen receptor T (CAR T) cells to specifically target surface antigen of cancer cells. Multiple targets are available for CAR T cell therapy including immunomodulatory antigens (PD-L1), overexpressed antigens (EGFR, HER2), aberrantly glycosylated proteins (MUC1). Suicide genes are being planned to be incorporated in CAR T cells to act as safety switch.

Differentiated CD4+ T cells play crucial role in providing beneficial immune responses against offending pathogens. Conversely, CD4+ T cells play various roles in the pathology of autoimmune inflammation. Effector CD4+ T cells, which were initially categorized as Th1 and Th2 cells by Mosmann et al. (3) have been expanded in the last 3 decades with the advent of Th17, Th9, Tfh, and Th22 cells. Importantly, CD4+ T cells not only initiate specific immune responses; subsets of CD4+ T cells have also been identified that are able to inhibit the initiation of immune reactions and even downregulate established immune responses. These CD4+ T cells are termed regulatory T cells (Tregs) and have, because of their role in the immunopathogenesis of autoimmune diseases and their potential use in therapeutic applications, become the focus of intensive research. IL-10-secreting Tregs have been denoted as Tr1 cells that do not express Foxp3. Naïve CD4+ T cells can find their niche in inflamed tissues in some autoimmune disorders, which would otherwise be limited between circulation and secondary lymphoid organs. However, allergic inflammation from Th2-mediated responses to environmental allergens and Th1-mediated immunity is responsible for the generation of multiple organ-specific experimental autoimmune diseases in animals.

Differentiation and regulation of CD4+ T cells depend on a plethora of factors including strength of antigen-antibody interaction, amount of co-stimulation, cytokines present in the milieu, expression of transcription factors and their interaction with histone modifiers. During the development of thymocytes CD154 co-stimulation plays prominent role to the TCR repertoire diversity. CD154 deficiency attenuates the sharing of TCRβ clone compared to the wild-type in T-cell dependent immune responses, leading to incorrect editing of T-cell clonotypes during the negative thymic selection (Fähnrich et al.). As the appreciation of T helper subset plasticity increases, it becomes even more important to characterize them. Distinctly opposite T helper subsets can express the same receptor, secrete a common cytokine and be regulated by the same transcription factor. Study by Huang et al., urge caution in using LAG3/CD49b co-expression as standalone markers for Tr1 cell identification as they can also be expressed by Foxp3+ Tregs and CD8+ T cells. Further studies would dissect the physiological relevance of the expression of these markers in different T cell subsets. Several studies have indicated the participation of distinct T helper subsets in the pathophysiology of inflammatory disorders (4). In a model of neuroinflammation, Th17 cells have been demonstrated to receive help from Tfh cells for the inflammatory B-cell response (Quinn et al.). B cells regulated by Tfh cells could move to the CNS and undergo class switching that correlated with disease severity (Quinn et al.).

Significant amount of information has been generated by researchers on the outcome of CD4+ T cells in various inflammatory disorders in both mice and humans. Behavior of CD4+ T cells, are regulated by internal metabolic properties. Lipid metabolites can act as regulators of immune responses. Alteration of steroids pathways can affect inflammation and be responsible for the pathophysiology of various diseases. The enzyme cholesterol 25-hydroxylase, which synthesizes 25-OHC, can enhance IL-27-induced T_r_1 cells (Vigne et al.). 25-OHC can negatively regulate Tr1 cells for the production of IL-10. T cell metabolism has been targeted for efficient cancer immunotherapy and altered effector T cell functions [(5), Roy et al.]. For the activation and proliferation of T cells, glucose provides the required energy (Roy et al.). Additional metabolites including lipids, ATP, nitric oxide, NAD also play crucial role in the differentiation of CD4+ T cells. Both mTOR and AMPK are sensors that regulate the metabolic checkpoints of T cell differentiation. mTOR inhibitors can attenuate glycolysis to induce memory T cell differentiation, while AMPK inhibitors decrease metabolism of fatty acids that in turn promote the differentiation of Th1 and Th17 cells. In contrast, activation of AMPK pathway can impart an analgesic effect in inflammatory pain by attenuating *Il1*β expression and blocking NF-κB activation (6). In naïve T cells, an increased AMP to ATP ratio is observed in the absence of TCR signaling leading diminished mTOR and sustained AMPK function (7). Interestingly during the lag phase of activated T cells, induced cytosolic calcium ions promote AMPK function in spite of decreased AMP level. However, in the growth phase of activated T cells increased ATP levels leads to sustained mTOR function. Depletion of the amino acids Trp and Arg can attenuate both the activation and function of effector T cells (5). Decreased level of oxygen and oxidative phosphorylation can increase PD-L1 expression on cancer cells. Activation of HIF-1 can induce effector properties of T cells by augmenting glycolysis and glutaminolysis. Other transcription factors that can act as metabolic checkpoints during T cell differentiation include BCL-6 (Tfh cell differentiation), IRF4 (Th2, Th17, Th9 cell differentiation), Foxo (Th9 cell differentiation), MYC (balance between Th17 and Treg cell differentiation). Drugs have been developed that target these various metabolic checkpoints to ameliorate various inflammatory diseases including Crohn's disease, ulcerative colitis, type 2 diabetes, rheumatoid arthritis, and chronic obstructive pulmonary disease. The activation of T cells is also mediated by essential trace metals including zinc. TCR signaling steps could potentially be altered by zinc (8). The zinc transporter Zip6, expressed on the surface of unstimulated T cells, is touted to be important bringing down the threshold of T cell activation. Vitamins also regulate the activation of T cells. Even though vitamin D blocks CD4+ T cell proliferation, it increases the number of CD4+CD25+Foxp3+ Treg cells. Therefore, T cell targeting via metabolic regulators represent exciting avenues for further investigations to regulate pathophysiology of inflammatory disorders and cancer.

Collectively, the articles published within the Research Topic highlight the emerging roles and underlying mechanisms of T cell differentiation and functions in tissue inflammation and their impact in the pathogenesis of inflammatory diseases. Based on the published work under this topic, it is further required to understand the functional dynamics of Th cell plasticity that lead to the ultimate outcome of immune-pathogenesis of diseases and lead to advancing our understanding for the immunological basis of diseases. The acquired immunological-based knowledge from the published articles will contribute further refined and novel immune strategies for inflammatory conditions.

## Author Contributions

RG and AA conceived, designed, and wrote the manuscript. All the authors read and approved the final manuscript for publication.

### Conflict of Interest

The authors declare that the research was conducted in the absence of any commercial or financial relationships that could be construed as a potential conflict of interest.
